# Structure of Plant Populations in Constructed Wetlands and Their Ability for Water Purification

**DOI:** 10.3390/plants14020162

**Published:** 2025-01-08

**Authors:** Junshuang Yu, Ling Xian, Fan Liu

**Affiliations:** 1 Core Botanical Gardens/Wuhan Botanical Garden, Chinese Academy of Sciences, Wuhan 430074, China; yujunshuang@outlook.com (J.Y.); xianling@wbgcas.cn (L.X.); 2 Changjiang Water Resources and Hydropower Development Group Co., Ltd., Wuhan 430010, China

**Keywords:** constructed wetlands, well-established plant species, plant community, wetland plants, wastewater treatment

## Abstract

In constructed wetlands (CWs) with multiple plant communities, population structure may change over time and these variations may ultimately influence water quality. However, in CWs with multiple plant communities, it is still unclear how population structure may change over time and how these variations ultimately influence water quality. Here, we established a CW featuring multiple plant species within a polder to investigate the variation in plant population structure and wastewater treatment effect for drainage water over the course of one year. Our results showed that the total species decreased from 52 to 36; however, 20 established species with different ecological types (emerged or submerged) remained with the same functional assembly for nutrient absorption, accounting for 94.69% of relative richness at the initial stage and 91.37% at the last state. The Shannon index showed no significant differences among the initial, middle, and last states. Meanwhile, regarding nutrient content, the total phosphorus (TP) concentration decreased by 57.66% at the middle stage and by 56.76% at the last state. Total nitrogen (TN) decreased by 50.86% and 49.30%, respectively. Chemical oxygen demand (COD) decreased by 36.83% and 38.47%, while chlorophyll a (Chla) decreased by 72.36% and 78.54%, respectively. Redundancy analysis (RDA) results indicated that none of the selected environmental variables significantly affected the species community except for conductivity. Our findings suggest that when utilizing multiple species for CWs, it is essential to focus on the well-established species within the plant community. By maintaining these well-established species, water purification in CWs can be sustained.

## 1. Introduction

As nature-based, environmentally friendly, effective, and convenient solutions for wastewater treatment, constructed wetlands (CWs) and their sustainability impacts have been studied for over 30 years [1,2,3]. CWs are efficient systems for removing various environmental pollutants found in wastewater [4]. Recent investigations indicate that CWs (hybrid type) are the most effective, achieving removal rates of 76%, 63%, and 72% for organic matter, nitrogen, and phosphorus, respectively [5]. In contrast, small CWs, based on data from 364 sites worldwide, demonstrate removal efficiencies of 50.2%, 50.5%, 65.2%, and 57.1% for total nitrogen (TN), total phosphorus (TP), chemical oxygen demand (COD), and ammonium nitrogen (NH_4_^+^-N), respectively.

Wetland plants are not only an important component of constructed wetlands, but they are also really crucial [5,6]. On one hand, they are an essential part of the wetland ecosystem and serve as the foundation for biodiversity [7]. On the other hand, they can provide oxygen to form aerobic, facultative and anaerobic micro-environments for other biological processes [8]. Additionally, wetland plants are vital for nutrient absorption, as they can take up nitrogen, phosphorus, and other nutrients from the water, thereby alleviating eutrophication and purifying sewage [9]. Some studies have sought to identify species suitable for use in CWs. For example, the wetland species *Cyperus articulatus* absorbed nearly 90% of organic matter and ammonium-nitrogen (NH_4_^+^-N) from CWs systems. The use of *Cyperus papyrus* resulted in nearly a 70% reduction in NH_4_^+^-N, 70% decrease in COD, and 50% reduction in TP [10].

Given the importance of wetland plants on CWs, most studies have primarily focused on individual plant species and their absorptive efficiency [11,12]. Only a few studies have examined the application of multiple plant species in CWs. In fact, the use of a species mix in CWs is considered beneficial for long term performance, as different species provide varying adaptive capacities to nutrient conditions over the short and long term [1]. Moreover, under natural conditions, it is also difficult for a single species to guarantee a stable plant stock over the long term. Different plants typically coexist in communities, representing a complex and dynamic process of change. The interactions involving competition and trade-offs among species ultimately determine the composition and diversity of species within these communities [13]. Meanwhile, environmental conditions also play a critical role in the dynamic changes of communities [14,15]. For example, fluctuations in water levels can facilitate the persistence of flood-tolerant plant populations, while nutrient-rich conditions may encourage the development of highly absorbed plant communities [16,17].

In CWs with several plant communities, it remains unclear whether the population structure will vary over time and how such variations may affect species richness and diversity. Furthermore, it is also uncertain how these variations ultimately influence water quality. What are the primary factors affecting these dynamics? Addressing these questions will provide valuable insights into the utilization of diverse plant species in the design and implementation of CWs. In this study, we established a constructed wetland (CW) featuring multiple plant species with several plant species in a polder to investigate changes in the structure of plant populations and changes in water quality over the course of a year. This evaluation aimed to assess the impact of changes in plant community composition on the overall effectiveness of water purification. We want to understand the natural dynamics of plant communities in CWs and whether these dynamics can sustain the effects of water purification.

## 2. Results

### 2.1. Plant Species Composition in CW

**Figure 1:**
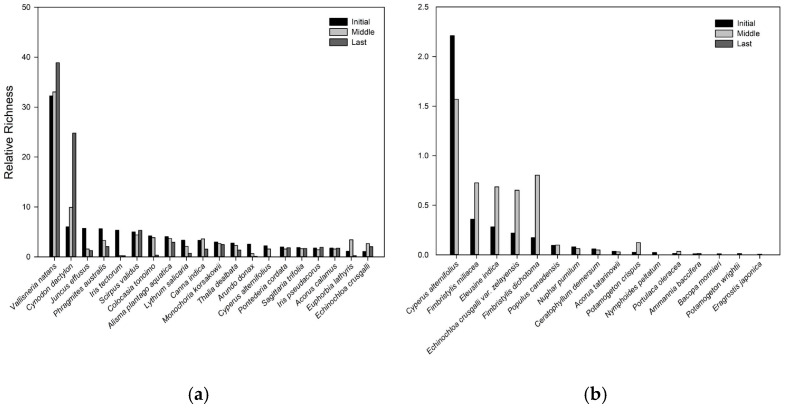
Relative richness of the 20 established species in the three states (**a**) and the 16 disappeared species in the last state (**b**) in CW.

Forty-seven (47) species were recorded after the CW was founded. Three months later, the species number increased to 50, while after one year, the total number of species decreased to 36 (Table S1). The 20 dominant species accounted for 94.69% of the relative richness at the initial stage, 85.09% at the middle stage, and 91.37% at the last state (Table S1, Figure 1a). All of the 20 species remained during the three stages except *Cyperus alternifolius* (Figure 1a), and 16 species had disappeared (Figure 1b). The first fivw dominant species are *Vallisneria natans* (initial: 32.25%, last: 38.88%), *Cynodon dactylon* (initial: 6.00%, last: 24.80%), *Juncus effusus* (initial: 5.70%, last: 1.26%), *Phragmites australis* (initial: 5.61%, last: 2.15%), and *Iris tectorum* (initial: 5.33%, last: 0.22%).

### 2.2. Species Diversity and Purification Effects of CW

**Figure 2:**
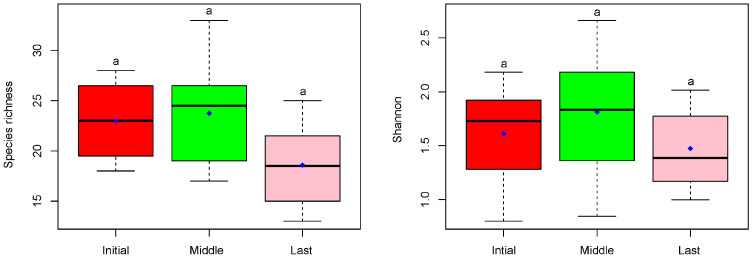
The comparison of Shannon index and the species richness among three stages (initial, middle, and last) in CW (N = 12 plots). The small letter ‘a’ indicated no the differences.

**Figure 3:**
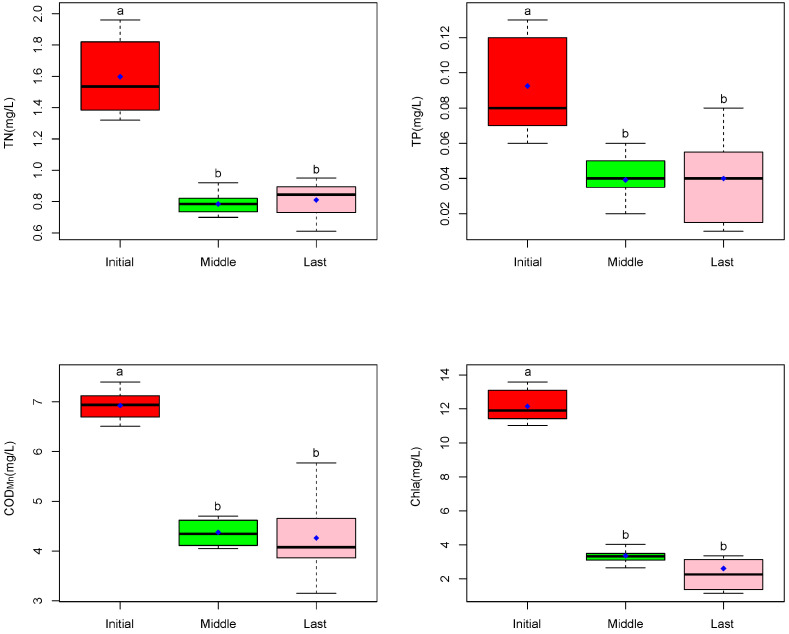
Nutrient content (TP, TN, COD, and Chla) among three states in CW (N = 12 plots). The small letter ‘a’ and ‘b’ indicated the differences.

Here, we used the Shannon index as the indicator for species diversity. The Shannon index increased in the first year but decreased in the last state compared to the initial. The one-way ANOVA indicated no significant differences among the three different stages (Figure 2). For the nutrient content, the TP concentration decreased by 57.66% in the first year and 56.76% in the second year, and TN decreased by 50.86% and 49.30%, respectively. COD decreased by 36.83% and 38.47%, while Chla decreased by 72.36% and 78.54% (Figure 3). The one-way ANOVA indicated significant differences among the three different stages (Figure 3).

### 2.3. The Relationship Between Environmental Data and Plant Species in CW

**Figure 4:**
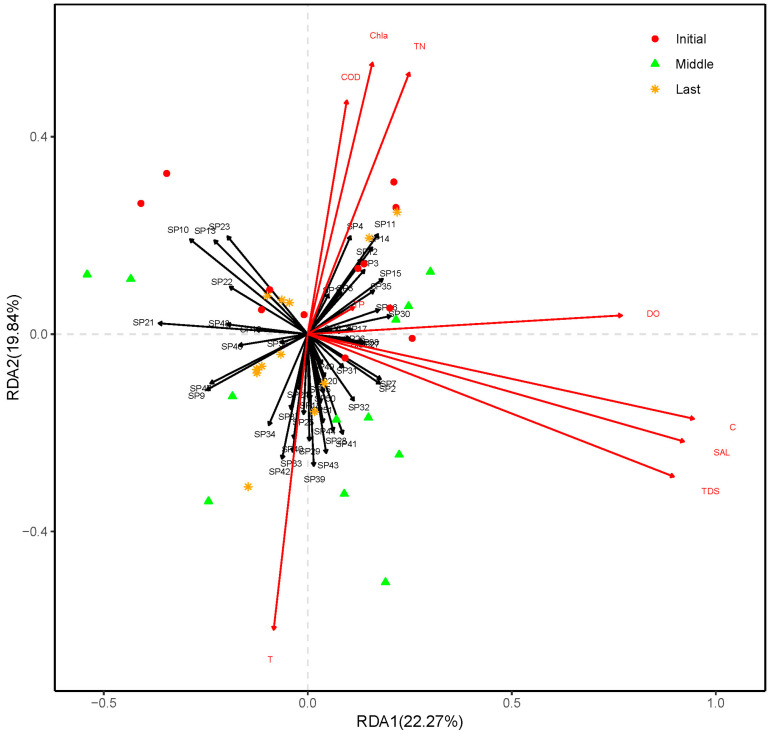
Redundancy analysis (RDA) (scaling = 2) of plant community with selected environmental variables.

The RDA axis 1 accounted for 22.27% of the variance, while the RDA axis 2, accounted for 19.84% of the variance. To assess the significance of environmental factors, an ANOVA was performed on the RDA model, and the results showed that the environmental factor C (*p* = 0.003) had a significant effect on species relative richness, while other factors such as T (*p* = 0.082), TDS (*p* = 0.649), SAL (*p* = 0.677), DO (*p* = 0.880), TN (*p* = 0.440), TP (*p* = 0.162), COD (*p* = 0.794), and Chla (*p* = 0.376) did not show significant effects (Figure 4).

## 3. Discussion

In our study, the natural variation of the plant community typically occurred in CW over time: Firstly, the Shannon index which indicated the variation of the evenness showed no differences over time and this may be attributed to the stability of the well-established plant species. Secondly, the total number of species decreased, but the well-established species within the plant community remained quantitatively stable. The species that ultimately disappeared contributed little to the overall population community. Those that remained quantitatively stable can be attributed to their greater adaptability and life history traits in relation to the CW environment. In particular, *V. natans* and *C. dactylon* were two species that had the highest relative abundance in the plant community. They occupy distinct ecological niches which enable them to minimize direct competition for sources. *V. natans* is a submerged species that is widely used in aquatic ecological restoration [18,19,20,21]. This species possesses strong asexual reproduction abilities through rhizomes and stolon, which confer interspecific competition advantage compared to other submerged plants [22,23]. Another beneficial adaptation of *V. natans* is that it spreads along the bottom of a waterbody and can compete for light [22,24]. Aquaculture water occasionally causes high nutrient load and low transparency in a waterbody, resulting in the disappearance of some of the submerged plants, such as *Potamogeton crispus* and *P. wrightii* in the CW, while *V. natans* tolerates lower light intensities, and the emerged species *P. natans* expands [24]. Unlike *V. natans*, *C. dactylon* is a species that can survive in a wide range of environments [25]. It is also the preferred choice for ecological restoration in riparian zones in China [26,27]. In our study, the heavy rains occurred in summer and the water level increased sharply due to the limited area. This species is more tolerant to flooding compared to the other species [28]. Besides, this species exhibits a high potential for reproductive ability, using both sexual reproduction through seeds and asexual reproduction via stolon, thereby facilitating the maintenance and expansion of its community and population [29]. In addition to these two dominant species, the core composition of other wetland plants exhibits remarkable adaptability and plasticity to aquatic conditions. For example, in Mozambique, Africa, Poaceae *Paspalidium obtusifolium* and Lentibulariaceae *Utricularia gibba* have comparable functions to *C. dactylon* and *V. natans*, respectively [30]. *P. obtusifolium* formed a compact floating mat that was rooted to the riverbed which enabled it to cope with the flooding conditions, and *U. gibba* had a higher ability in deep water [30]. Besides those, *P. australis* demonstrates greater plasticity in shoot length [31], while *J. effusus* and *E. crus*-*galli* exhibit high tolerance to flooding within a short time [32,33]. All of these factors contribute to the stability of the well-established species within the community over time.

Similar to the changes in species, the diversity of wetland plants also exhibited slight variation over one year and a half. This may be attributed to the stability of well-established species in CW, which accounted for nearly 90% of species abundance. Although there was a reduction in species numbers, the disappearing species contributed very little to the overall diversity, resulting in no significant changes in community diversity patterns over time in our study. Despite the absence of significant changes in diversity, wetland plants substantially impacted the water quality purification of aquatic systems. There has been a sustained and significant decrease in total nitrogen and total phosphorus in the water, which is linked to the plants’ inherent absorption capabilities. After the pumped aquaculture water is introduced into the central pond, the plants absorb nutrients from the water through their roots and leaves to support their growth and population expansion. Although our study did not give the exact results of the absorption efficiency of the specific species respectively, some of the former studies have investigated them. For instance, *V. natans* achieves nutrient removal efficiencies of 65% for total nitrogen, 52% for total phosphorus, and 45% for COD [34]. Similar findings involve wetland plants such as *C. dactylon*, *P. australis*, and *J. effusus*, along with all other well-established species in CWs, which exhibit nutrient absorption capacities of 65%, 60%, and 55%, respectively [35,36,37,38]. Meanwhile, the significant reduction in algal abundance in the water may be related to the decrease in total phosphorus levels, as the variation in the number of phytoplankton mainly depends on light and nutrient availability [39]. Given that the conditions, such as light and climate, were consistent in the same season (August 2023 and August 2024), the significant decrease in chlorophyll a content primarily results from the decline in total phosphorus. Overall, plants exhibit a significant absorption effect on nutrients based on their inherent capabilities.

Wetland plants play an important role in nutrient absorption and studies indicate that the aquatic environment may also influence plant communities [7]. However, the results of this study show that environmental factors did not significantly influence the structure of the plant community. This may be explained by the species coexistence theory. According to the theory, under moderate disturbance conditions, changes in plant communities may not be limited by environmental factors, but rather by the competition among species themselves [40]. There are pairwise interactions and higher-order interactions among species, and the ultimate result of competition is that a few core species survive and maintain their population characteristics [40]. This study provides new evidence for this theory and suggests that changes in CW plant communities are the result of competition among the plants themselves. Although the aquaculture effluent has characteristics such as high nutrients, RDA analysis results show that nutrients do not influence population dynamics. Through the changes observed in plant communities and the results of diversity, we also find that the changes in plant communities in this CW may arise from the competition among the plants. For example, species occupying the same ecological niches will compete for the same sources and one of the species may disappear finally [13]. For example, *C. dactylon* and *Cyperus alternifolius* occupy the same ecological niches within the CW. However, *C. dactylon* shows stronger population expansion capabilities and greater water tolerance than *C. alternifolius*, ultimately leading to the disappearance of *C. alternifolius*.

## 4. Materials and Methods

### 4.1. Study Site and Experimental Design

**Figure 5:**
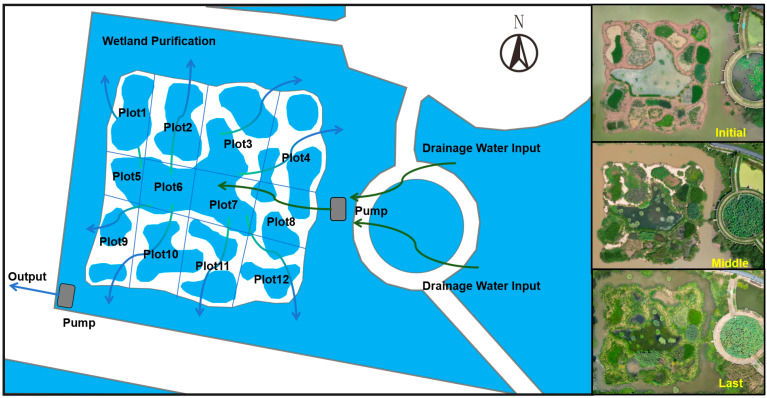
The design and information of the constructed wetland of the polder.

The polder is located in Wuhu farmland in Wuhan, covering a 5 5-acre area in central China. On March 2023, at the initial state of the polder, 24 native species were recorded and 23 common species were introduced and transplanted into the polder (Table S1). Among these 47 species, 4 of them are submerged (*Vallisneria natans*, *Hydrilla verticillata*, *Ceratophyllum demersum*, *Potamogeton crispus*) while 4 of them are floating species (*Nymphaea tetragona*, *Nuphar pumilum*, *Nymphoides peltata*, *Marsilea quadrifolia*). Most of them have been used for wetland ecological restoration and landscape architecture (Table S1). The polder was divided into 12 parts, creating 12 plots. Drainage water was pumped into the middle pond of the polder, and then flowed through the polder in 8 different directions (Figure 5). The 47 species were transplanted randomly among the 12 plots. Finally, the CW was built for drainage water.

### 4.2. Data Collection

For each plot, all species were recorded and the total number of each species was counted. Meanwhile, environmental data including temperature (T), conductivity (C), total dissolved solids (TDS), salinity (SAL), dissolved oxygen (DO), total nitrogen (TN), total phosphorus (TP), COD, and chlorophyll a (Chla) were also collected from each plot. T, C, TDS, and SAL were recorded by a YSI pro-plus multi-probe. DO was recorded by YSI pro-ODO meter. The TN, TP, COD, and Chla of the water were measured and recorded in the lab. TP and TN were measured using UV spectrophotometry [41,42]. Chla content was measured by filtering 20 L water and then transferring to ethanol for approximately 24 h in the dark. The absorbance was subsequently measured by a spectrophotometer [41]. COD was measured by spectrophotometer with the potassium dichromate method [41]. All the data including the plant species and the environmental variables were collected during the initial stage (initial: March 2023), 3 months after CW construction (middle: Second: August 2023), and one year later (last: August 2024).

### 4.3. Data Analysis

The species richness of each plot was defined as the total number of species and the relative evenness was calculated for each plot. A diversity was also calculated and illustrated by the Shannon index. To assess the species and the purification effects of the CW over time, the species, the species richness, and α diversity, along with the environmental data, were compared among the three sampling times (initial, middle, and last). One-way ANOVA was used to analyze differences among the periods. Tukey HSD was applied for single-step (default) multiple comparisons of means between sampling times at a 95% family-wise confidence level. In addition, to investigate species–environment relationships, redundancy analysis (RDA) was used to quantify the contribution of local environment factors on species composition for the whole species dataset [42]. All of the data were calculated by R 4.3.2.

## 5. Conclusions

In summary, previous research on CW utilizing wetland plants often focused on single plant or species pairs. Our findings suggest that when employing multiple species in CW, especially landscape wetlands, it is appropriate to focus on the well-established species within the plant community. By maintaining these well-established species, water purification in the CW can be effectively sustained. However, since this research was conducted over just one and a half years, it may not fully capture the long-term dynamics of the population. Ongoing studies will continue to build on the current findings.

## Data Availability

Data are contained within the article.

## Supplementary Materials

The following supporting information can be downloaded at: https://www.mdpi.com/article/10.3390/plants14020162/s1, Table S1: The list and variation of the originally already existing and added species among the initial, middle, and last cultivated stages.
