# Supplementary material for: Structure of Plant Populations in Constructed Wetlands and Their Ability for Water Purification
Source: Plants (Basel). 2025 Jan 8;14(2):162. doi: 10.3390/plants14020162 (PMC11768403; doi:10.3390/plants14020162)
Supplement: Supplementary file 1 [file plants-14-00162-s001.zip › plants-3342281-supplementary.pdf]

Table S1. The list and variation of the originally already existing and added species among the initial, middle and last cultivated stages

Family	Genus	Species	Already existing species	Added species	Initial	Middle	Last
Alismataceae	*Alisma*	*Alisma plantago aquatica*		+	+	+	+
	Sagittaria	*Sagittaria trifolia*		+	+	+	+
Amaranthaceae	*Alternanthera*	*Alternanthera philoxeroides*	+		+	+	+
Araceae	*Acorus*	*Acorus calamus*		+	+	+	+
		Acorus tatarinowii	+		+	+	
	Colocasia	*Colocasia tonoimo*		+	+	+	+
Cannaceae	*Canna*	*Canna indica*		+	+	+	+
Ceratophyllaceae	*Ceratophyllum*	*Ceratophyllum demersum*		+	+	+	
Cyperaceae	*Carex*	*Carex sp*	+		+	+	+
	Cyperus	*Cyperus alternifolius*		+	+	+	
		Cyperus difformis	+		+	+	+
		Cyperus iria	+		+	+	+
		Cyperus microiria				+	+
	Fimbristylis	*Fimbristylis dichotoma*	+		+	+	
		Fimbristylis miliacea	+		+	+	
	Scirpus	*Scirpus validus*		+	+	+	+
Gentianaceae	*Nymphoides*	*Nymphoides peltatum*		+	+		
Gramineae	*Arundo*	*Arundo donax*		+	+	+	+
	Cynodon	*Cynodon dactylon*	+		+	+	+
	Echinochloa	*Echinochloa caudata*	+		+	+	+
		Echinochloa crusgalli	+		+	+	+
		Echinochloa crusgalli var. zelayensis	+		+	+	

	Eleusine	*Eleusine indica*	+		+	+	
	Eragrostis	*Eragrostis japonica*				+	
	Euphorbia	*Euphorbia lathyris*	+		+	+	+
	Pennisetum	*Pennisetum alopecuroides*	+		+	+	+
	Phragmites	*Phragmites australis*		+	+	+	+
	Setaria	*Setaria faberi*	+		+	+	+
	Triarrhena	*Triarrhena sacchariflora*	+		+	+	+
Hydrocharitaceae	*Hydrilla*	*Hydrilla verticillata*		+	+	+	+
Hydrocharitaceae	*Vallisneria*	*Vallisneria natans*		+	+	+	+
Iridaceae	*Iris*	*Iris pseudacorus*		+	+	+	+
		Iris tectorum		+	+	+	+
Juncaceae	*Juncus*	*Juncus effusus*		+	+	+	+
Leguminosae	*Aeschynomene*	*Aeschynomene indica*	+		+	+	+
Lemnaceae	*Lemna*	*Lemna minor*	+		+	+	+
Lythraceae	*Ammannia*	*Ammannia baccifera*	+		+	+	
	Lythrum	*Lythrum salicaria*		+	+	+	+
Marantaceae	*Thalia*	*Thalia dealbata*		+	+	+	+
	Marsilea	*Marsilea quadrifolia*		+	+	+	+
Nymphaeaceae	*Nuphar*	*Nuphar pumilum*		+	+	+	
Nymphaeaceae	*Nymphaea*	*Nymphaea tetragona Georgi*		+	+	+	+
Onagraceae	*Ludwigia*	*Ludwigia prostrata*	+		+	+	+
Polygonaceae	*Polygonum*	*Polygonum hydropiper*					+
		Polygonum lapathifolium var. salicifolium	+		+	+	+
Pontederiaceae	*Monochoria*	*Monochoria korsakowii*		+	+	+	+
Pontederiaceae	*Pontederia*	*Pontederia cordata*		+	+	+	+

Portulacaceae	*Portulaca*	*Portulaca oleracea*	+	+	+
Potamogetonaceae	*Potamogeton*	*Potamogeton crispus*	+	+	+
		Potamogeton wrightii			+
Salicaceae	*Populus*	*Populus canadensis*	+	+	+
Scrophulariaceae	*Bacopa*	*Bacopa monnieri*			+
